# Development of Auer bodies from giant inclusions associated with rough endoplasmic reticulum in acute promyelocytic leukemia

**DOI:** 10.1097/BS9.0000000000000163

**Published:** 2023-05-30

In the article titled “Development of Auer bodies from giant inclusions associated with rough endoplasmic reticulum in acute promyelocytic leukemia,” published on pages 111-117, Volume 5, Issue 2 of Blood Science, the figure caption for figure 6 has been changed for accuracy in the online version.

“Figure 6. Mature Auer bodies. (a) and (b) show myeloperoxidase reactivity of promyeloblasts. (a) illustrates Auer bodies (arrowheads) – one of them irregular in shape – and granules in the perinuclear space (arrows), N, nucleus (unstained) ×10k; (b) shows longitudinal sections (arrows) and cross-sections (arrowheads) of Auer bodies in promyeloblasts, ×5k; (c), an Auer body has a double-crystalized center and an enveloping membrane (arrowhead) similar to that of the nuclear membrane (arrow), ×15 k; (d), an Auer body with a single crystallized center (arrows), ×15 k.

In addition, figure 6 has been replaced with an updated version online.
